# Benefits of a 3-month cycle of weekly virtual museum tours in community dwelling older adults: Results of a randomized controlled trial

**DOI:** 10.3389/fmed.2022.969122

**Published:** 2022-08-16

**Authors:** Olivier Beauchet, Jacqueline Matskiv, Kevin Galery, Linda Goossens, Constance Lafontaine, Kim Sawchuk

**Affiliations:** ^1^Departments of Medicine and Geriatrics, University of Montreal, Montreal QC, Canada; ^2^Research Center of the Geriatric University Institute of Montreal, Montreal QC, Canada; ^3^Division of Geriatric Medicine, Department of Medicine, Sir Mortimer B. Davis Jewish General Hospital and Lady Davis Institute for Medical Research, McGill University, Montreal QC, Canada; ^4^Lee Kong Chian School of Medicine, Nanyang Technological University, Singapore, Singapore; ^5^Education and Wellness Department of the Montreal Museum of Fine Arts, Montreal, QC, Canada; ^6^Faculty of Arts and Science, Concordia University, Montreal, QC, Canada

**Keywords:** older adults, social isolation, art, museum, wellbeing, quality of life, frailty

## Abstract

**Background:**

Museums can be instrumental in fostering social inclusion and may improve the overall health of the older population. Over the course of the 2019 coronavirus pandemic, many older adults suffered as a result of confinement measures, which may have accelerated the processes that lead to physical frailty and increased mental health risks. This study aims to examine whether a 3-month cycle of weekly virtual tours of the Montreal Museum of Fine Arts (MMFA) may have improved feelings of social inclusion, wellbeing and quality of life, and reduced physical frailty in older adults living within the community of Montreal.

**Methods and design:**

A total of 106 older adults, who were community-dwellers living in Montreal (Quebec, Canada), were recruited for a randomized controlled trial in two parallel groups (intervention with *n* = 53 *vs* control with *n* = 53) between January and April 2022. The intervention consisted of a 3-month cycle of weekly virtual museum tours of the MMFA. Social isolation, wellbeing, quality of life and frailty were evaluated using validated scales that were assessed on a web platform at baseline (M0) and after 3 months (M3) in the intervention group. The control group completed the same assessment according to the same schedule. The outcomes were the mean scores at M0 and M3, and changes in mean scores between M0 and M3.

**Results:**

The intervention group showed significant improvements in their social isolation, wellbeing, quality of life and frailty scores when compared to the control group, the highest benefits being observed with frailty.

**Conclusion:**

The results suggest that the 3-month cycle of weekly virtual MMFA tours may improve social inclusion, physical and mental health in community-dwelling older adults living in Montreal.

**Trial registration:**

https://clinicaltrials.gov/ct2/show/NCT05046288, identifier NCT05046288.

## Background

Over the past 2 years of the coronavirus disease 2019 (COVID-19) pandemic (1), physical distancing was deployed as a preventive public health measure to reduce the transmission of the severe acute respiratory syndrome coronavirus 2 (SARS-CoV-2). This physical distancing had at least two unintended consequences for older adults. First, these measures often deprived older people of face-to-face access to social activities and social networks, which often led to an increase in their social isolation (2). Second, in many countries, such as Canada, there was an increased demand for access to the health care system and community resources, which could not always be met (3, 4). This combination of an increase in social isolation and increased pressure on the healthcare system and community organizations often led to a degradation in physical and mental health, making older adults frail and increasing their risk of adverse outcomes (5). For instance, research suggests that older adults who experience social isolation are at a greater risk for incident morbidities, which can contribute to greater physical frailty and even premature death (5, 6). Conversely, physical frailty itself may also increase social isolation (5). Moreover, it has been demonstrated that older adults' wellbeing and quality of life are impacted negatively by both social isolation and physical frailty (5–8). This highlights the need for innovative interventions that promote the social inclusion of older adults, especially in the wake of 2 years of the COVID-19 pandemic.

Systematic reviews of quantitative studies have attempted to evaluate the effectiveness of interventions designed to increase the social inclusion, and sense of connectedness, of older adults experiencing social isolation (8–15). Due to the heterogeneity of interventions and their results, to date, there has been no conclusive evidence on the effectiveness of specific strategies to increase social inclusion in this population.

Further research is required to determine what “works” to improve social inclusion. These systematic reviews do, however, point to three key characteristics of effective interventions. First, *group activities* have a greater effect than those performed alone (8–12). Second, engaging participants in *goal-oriented endeavors*, rather than in passive activities with no explicit purpose, appears to be more effective in increasing feelings of social inclusion (12–15). Third, activities that include a creative component (such as arts-based activities) evoke positive emotions that are beneficial (15). Moreover, cultural interventions predicated on any type of arts-based activity have been shown to have beneficial effects that may improve people's quality of life (16, 17).

A socially-inclusive society enables all to remain engaged in collective daily life for as long as possible as they age (18). The concept of social inclusion implies on-going, meaningful participation in society. Providing occasions and places where individuals may participate in shared activities are key attributes of an inclusive society. Because they offer a variety of opportunities to participate meaningfully in arts-based group activities - from guided tours to lectures and workshops - museums may fulfill such a role, fostering a sense of social inclusion. Indeed, the potential of museums to improve the social inclusion of older adults experiencing social isolation has been demonstrated in a British study on “museums as spaces for wellbeing.”^1^ Since 2015, a participatory, arts-based workshop series has been offered by the Montreal Museum of Fine Arts (MMFA, Montreal, Quebec, Canada) (19). In an examination of this program, it was demonstrated that an intervention involving art creation in a group setting at the MMFA improved the wellbeing, quality of life and health condition of community-dwelling older adults in Montreal (19). Building on this initial study, in 2019, we then co-developed an arts-based activity with the MMFA, consisting of weekly guided tours carried out over a 3-month cycle. Because of physical distancing requirements during the COVID-19 pandemic, these guided tours were adapted into virtual guided tours. The impacts and effects of such virtual tours on older adults experiencing social isolation had never been examined. We hypothesized that weekly virtual MMFA tours could reduce social isolation and improve the wellbeing, quality of life and health condition, including the physical frailty, of older adults living in Montreal. This study thus aims to examine whether a 3-month cycle of weekly virtual tours of the MMFA may have improved feelings of social inclusion, wellbeing and quality of life, and reduced physical frailty in older adults living within the community of Montreal.

## Methods

### Design

The study was a uni-center (*Center Intégré Universitaire de Santé et des Services Sociaux du Center-Sud-de-l'ile-de-Montréal*, Quebec, Canada) randomized controlled trial (RCT) in two parallel groups (*i.e.*, intervention group, which participated in virtual MMFA tours *vs* control group, which did not participate in virtual MMFA tours). The control group participants were asked to avoid participation in any arts-based activity 3 months ahead of the study and over the 3-month period of the study itself. Participants were aware of the intervention, and therefore not “blinded” due to the nature of the intervention, which required their explicit commitment to a 3-month cycle of weekly virtual MMFA tours. All staff members of the research team involved in the RCT phases (*i.e.*, recruitment, assessment, and follow-up) were blinded to the allocation of intervention, except one staff member who was responsible for the randomization list. Participants were randomly allocated into intervention and control groups by block randomization with block sizes of 1:1. Randomization lists were established using the N'Query randomization software. This RCT is registered on the ClinicalTrials.gov website (project number NCT05046288) and followed the CONSORT guidelines for RCTs (20).

### Population

**Figure 1:**
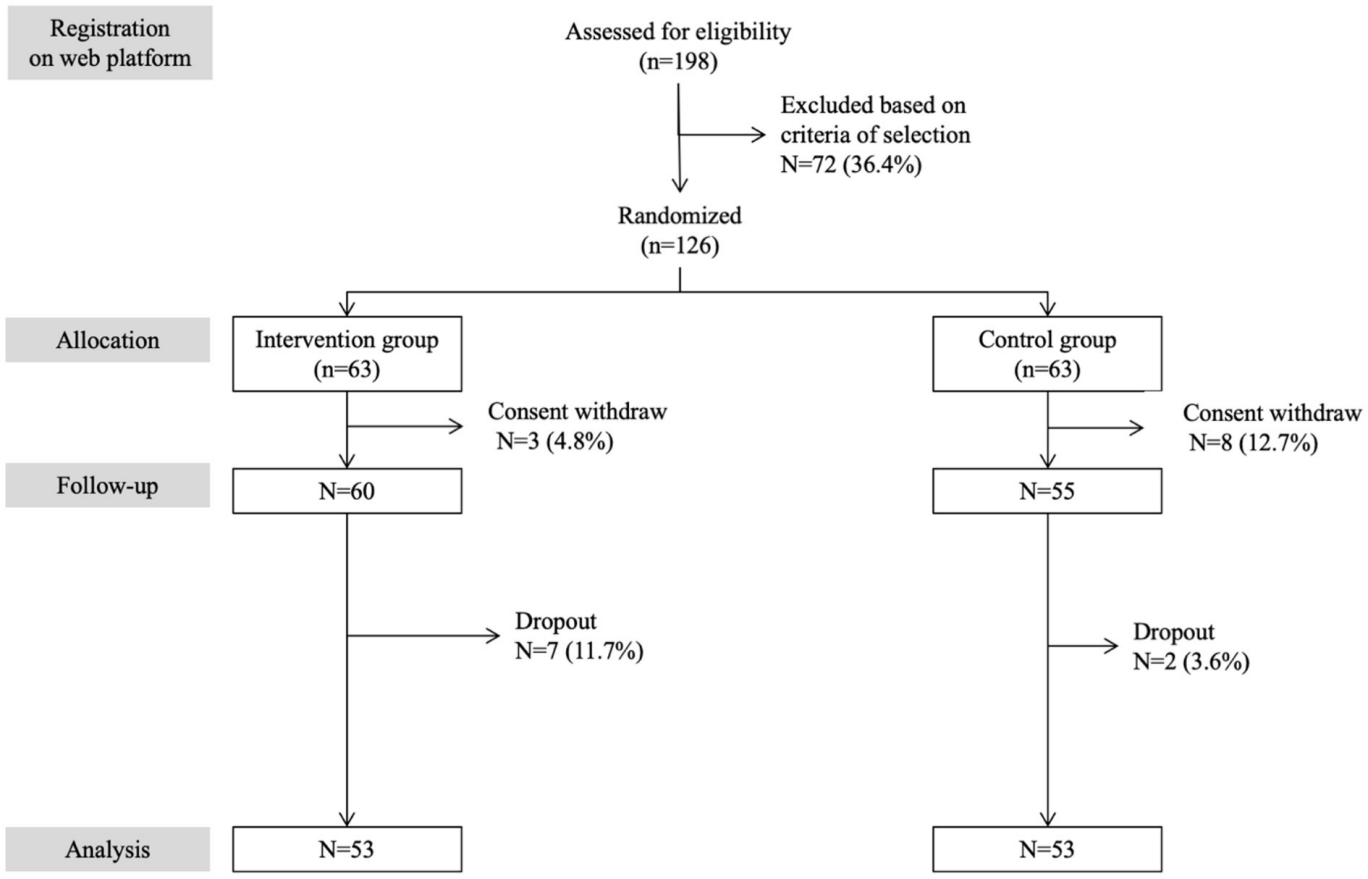
Consort flow diagram detailing selection and follow-up of participants in the RCT.

A total of 106 participants were enrolled and completed the full study between January and April of 2022. The inclusion criteria were as follows: aged 65 and over with a life expectancy over 6 months (according to a free software that incorporates socio-demographic, cardio-vascular risk factors, physical activity and income characteristics)^2^, experiencing social isolation as defined by the 11-item Duke Social Support Index (DSSI) score ≤ 28/33 (see footnote 1), living at home in the urban area of Montreal (Quebec, Canada), able to communicate and write in the language of the recruitment center (*i.e.*, French, English or Chinese) and able to consent to participate in the study. The participants were screened using information provided by community associations in Montreal. They were informed that a clinical study on the effects of weekly virtual MMFA tours on social inclusion, wellbeing, quality of life and health condition was launched by the MMFA in partnership with the Research Center of the Geriatric University Institute of Montreal (CRIUGM; Montreal, Quebec, Canada) and that the MMFA and CRIUGM were recruiting participants. Potential participants registered *via* their neighborhood associations on the CRIUGM website. If they needed more information on the study, they had the option of calling someone at the CRIUGM. A total of 198 individuals registered on the web platform. They were contacted by a staff member of the research team for an interview by phone. During this phone call, the objective of the study and its procedures were explained and the selection criteria for participants were validated. Following these calls, 72 (36.4%) of potential participants were excluded because of the selection criteria and 126 (63.6%) individuals were enrolled, signed the consent form, and randomized into intervention (*n* = 63) and control (*n* = 63) groups. Among them, 11 (8.7%; 3 in the intervention and 8 in the control group) withdrew their consent before the baseline assessment. In total, 115 (91.3%) participants (60 in the intervention group and 55 in the control group) underwent the full baseline assessment. Seven (6.1%) participants in the intervention group and 2 (1.7%) in the control group dropped out over the 3-month period of the study. There was no significant difference in baseline characteristics between the group of participants who withdrew their consent and dropped out, and the group of those who completed the study (data not shown). Figure 1 shows a flow diagram detailing participant selection and follow-up in the RCT.

### Intervention

The intervention consisted of a 3-month cycle of weekly virtual MMFA guided tours. Each visit was performed with a group of 6 to 8 participants and a trained guide, for a total of 8 groups. Participants met once a week over the 3-month period and participated in a 45-minute virtual guided tour *via* the videoconferencing platform Zoom, using their own digital device. An additional 15-minute period dedicated to informal discussion (to allow for more socializing) was proposed after each tour (this extra discussion time was optional).

Regardless of the topics or the themes covered, each visit was standardized and separated into three consecutive phases: presentation of the visit objectives, a dialogic-style tour with trained museum guides, and an open-ended discussion after the tour. The tour content consisted of a combination of images of artworks (e.g., paintings, sculpture, decorative pieces), live discussions animated by the tour guides, ancillary information on the artworks or artists from tour guides, and pre-recorded videos about specific works or artists. The amount and difficulty of information presented to participants was increased each month over the 3-month cycle of guided museum tours.

Each weekly guided tour was unique and led by a single museum guide. One guide was assigned to each group of 8 participants for all 12 visits. Additionally, one member of the research team was assigned to each group to oversee the research components of the tour, participate in a virtual ethnography, manage participants' potential technical issues (e.g., difficulties connecting to the Zoom meeting, issues with sound, display) and assist in the presentation of the visual content (images and videos).

### Assessment

#### Baseline assessment

The baseline assessment was performed at participants' place of living *via* a web platform using standardized procedures and digital questionnaires before (M0) the first tour with support by phone if needed. Both the intervention and control groups performed the baseline assessment. The participants' socio-demographic characteristics (i.e., age, sex, ethnicity, place of living) were recorded. Social isolation was assessed using the 11-item Duke Social Support Index (DSSI) (21). The index comprises two subscales: social interaction (i.e., frequency of interactions) and subjective support (i.e., satisfaction with emotional support provided). DSSI scores range from 11 to 33, with higher scores indicating higher levels of social inclusion. Wellbeing was assessed using the Warwick-Edinburgh Mental Wellbeing Scale (WEMWBS) self-administered questionnaire (22), which is composed of 14 positively-worded items and produces scores ranging from 14 (i.e., none of the time) to 70 (i.e., all the time). EuroQol-5D (EQ-5D) was used to assess health-related quality of life (23). This tool is composed of a questionnaire examining physical health issues, with scores ranging from 0 (i.e., no issue) to 25 (i.e., worst issues), and a visual analog scale (VAS) assessing self-perceived health, ranging from 0 (i.e., worst health imaginable) to 100 (i.e., best health imaginable). Physical and mental frailty was assessed using the Center of Excellence Self-AdMinistered questionnaire (CESAM) (19, 24). Using 20 close-ended questions, CESAM examines different subdomains of mental and physical health: weight loss; polypharmacy (i.e., number of therapeutic classes taken on a daily basis ≥ 5); vision, hearing and memory problems; home support; activities of daily living (ADL) and instrumental activities of daily living (IADL) (25, 26); mood; practice of regular physical activity; and history of falls in the past 12 months. CESAM was filled out by the participants themselves under the supervision of Principal Investigator representatives. The total health frailty score ranges from 0 (i.e., best health condition) to 18 (i.e., worst health condition).

#### Follow-up assessments

DSSI, WEMWBS, EQ-5D and CESAM questionnaires were repeated after the twelfth (M3) tour in intervention and control groups. Like the baseline assessment, all questionnaires were completed online at participants' place of living with support by phone if needed. After the M3 assessment, the participants in the control group were offered a complimentary virtual MMFA tour to compensate them for their compliance and restraint from art and museum-going activities during the period of the study.

### Outcome measures

The primary outcome was captured by the DSSI score. The secondary outcomes were captured by the scores of WEMWBS, EQ-5D and CESAM. For each outcome, the mean score at M0 and M3, and changes in mean score between M0 and M3 {using the formula [(score M3 – score M0) / (score M3 + score M0) /2] × 100} were used (19).

### Ethical considerations

Participants were included after giving written, informed consent for research. The study was approved by the *CIUSSS Center-Sud-de-l'Île-de-Montréal* (Quebec, Canada) Research Ethics Committee (# 2022-1338 – CÉR VN 21-22-08).

### Statistics

Means, standard deviations (SD), frequencies and percentages are used to describe participants' characteristics. Inter- and intra-group comparisons were performed using unpaired or paired *t*-tests, and Chi-squared tests, as appropriate. Multiple linear regressions were used to examine the association between variations of each questionnaire's score (used as dependent variables with separated models for each score) and the intervention (used as independent variables), were adjusted according to participants' baseline characteristics. *P*-values less than 0.05 were considered statistically significant for linear regressions. All statistics were performed using SPSS (version 23.0; SPSS, Inc., Chicago, IL).

## Results

As shown in Table 1, there was no significant difference between groups for participants' baseline characteristics, except for sex and ethnicity. There were fewer females and Caucasians in the intervention group compared to the control group (*P* ≤ 0.013). There were significant greater mean scores for DSSI, EQ-5D and CESAM (*P* ≤ 0.001) at M3 compared to M0 in the intervention group (Table 2). There was only a trend (*P* = 0.059) for greater WEMWBS mean scores in the intervention group. No significant change in all scales' scores between M0 and M3 was found in the control group. Inter-group comparisons showed that DSSI, WEMWBS, EQ-5D and CESAM mean scores were significantly higher in the intervention group compared to the control group at M3 (*P* < 0.033), while significant difference was found at M0. Table 3 shows that participation in weekly virtual MMFA tours was significantly associated with improvements in all scales (*P* ≤ 0.012).

**Table 1:** Baseline participant characteristics (*n* = 106).

	**Participants**		P-Value^*^
	**Control** **(*n =* 53)**	**Intervention (*n =* 53)**	
Age (years), mean ± SD	74.3 ± 5.1	75.0 ± 4.6	0.458
Female, *n* (%)	48 (90.6)	38 (71.7)	**0.013**
Caucasian, *n* (%)	53 (100)	45 (84.9)	**0.003**
Place of living home, *n* (%)	47 (88.7)	46 (86.8)	0.696
Living alone, *n* (%)	33 (62.3)	36 (67.9)	0.838
Home support^†^, *n* (%)	1 (1.9)	3 (5.7)	0.308
ADL score (/6)^‡^, mean ± SD	5.8 ± 0.5	5.6 ± 0.8	0.233
IADL score (/4)^ǁ^, mean ± SD	3.9 ± 0.2	3.9 ± 0.2	1.000
Polypharmacy^§^, *n* (%)	38 (71.7)	43 (81.1)	0.253
SARS-CoV2 status, *n* (%)			
Never infected	4 (7.5)	4 (7.5)	1.000
Vaccinated	51 (96.2)	53 (100.0)	0.153
Happy mood^¶^, *n* (%)	23 (43.4)	27 (50.9)	0.436
Practice of physical activity^**^, *n* (%)	46 (86.8)	37 (69.8)	0.034
History of falls in the past 12 months, n (%)	16 (30.2)	14 (26.4)	0.666

*SD, Standard deviation; ADL, Activities of daily living; IADL, Instrumental activities of daily living;*

* *Comparison based on unpaired t-tests or chi-squared, as appropriate;*

† *Receiving help from family, friend or professional for daily living activities;*

‡ *Ranging from 0 (dependent) to 6 (independent);*

ǁ *Ranging from 0 (non-autonomous) to 4 (autonomous);*

§ *Number of therapeutic classes taken daily ≥ 5;*

¶ *Answer to the question “How do you feel today?” with three possible answers, including unhappy, happy, neither one nor the other;*

** *Regular physical activity (walking, bicycle, etc.) at least 1 h per week in the past month; P-value significant fixed < 0.0035 because of multiple comparisons (n = 14)*.

**Table 2:** Comparisons of mean values of scales assessing social isolation, wellbeing, quality of life and frailty between control and intervention groups (*n* = 106).

	**Participants**						***P*** **-value between group comparisons^¶^**	
	**Control (*****n =*** **53)**			**Intervention (*****n =*** **53)**			**M0**	**M3**
	**M0**	**M3**	***P*** -Value^*^	**M0**	**M3**	***P*** -Value^*^		
11-item Duke Social Support Index (/33)^†^, mean ± SD	25.3 ± 2.7	25.7 ± 3.2	0.240	24.6 ± 3.1	27.1 ± 3.2	**≤0.001**	0.205	**0.033**
Warwick-Edinburgh Wellbeing scale (/70)^‡^, mean ± SD	54.6 ± 6.3	53.6 ± 4.7	0.359	56.6 ± 6.4	58.3 ± 5.5	0.059	0.109	**≤0.001**
EQ-5D, mean ± SD								
Questionnaire score (/25)^ǁ^	6.6 ± 1.6	7.0 ± 2.0	0.052	6.8 ± 2.0	8.6 ± 2.1	**≤0.001**	0.747	**≤0.001**
Visual analog scale (/100)^§^	78.5 ± 11.5	78.5 ± 14.2	0.992	77.5 ± 14.2	86.6 ± 10.5	**≤0.001**	0.686	**0.001**
Frailty Score (/18)^#^, mean±SD	6.2 ± 3.3	5.4 ± 2.4	0.177	6.7 ± 4.0	2.1 ± 1.0	**≤0.001**	0.464	**≤0.001**

*SD, Standard deviation; EQ-5D, EuroQuol 5D; M, Month; M0, baseline assessment before intervention; M3, Assessment at the end of the 3-month intervention period;*

* *Comparisons based on paired t-test;*

† *Ranging from 11 (social isolation) to 33 (absence of social isolation);*

‡ *Ranging from 14 (i.e., none of the time) to 70 (i.e., all the time);*

ǁ *Ranging from 0 (no problem) to 25 (unable to do);*

§ *Ranging from 0 (the worst health condition) to 100 (the best health condition);*

# *Mean score calculated from computerized self-administered questionnaire composed of 20 questions providing a score ranging from 0 (vigorous) to 18 (severe frailty);*

¶ *Comparison based on unpaired t-tests; significant P-values in bold fixed at < 0.003 because of multiples comparisons (n = 15)*.

**Table 3:** Multiple linear regressions showing the association of intervention (i.e., 3-month period of virtual guided tour, independent variable) and changes in mean score between baseline assessment and end of intervention for 11-item Duke Social Support Index, Warwick-Edinburgh Wellbeing scale, EuroQol-5D and Center of Excellence Self-AdMinistered questionnaire scores adjusted for baseline participant's characteristics (*n* = 106).

Change in mean score between baseline assessment and the end of intervention^*^	**Effect of intervention**		
	**β**	**[95%CI]**	***P*** **-Value**
11-item DSSI	10.58	[5.44–15.72]	**≤0.001**
WEMWBS score	7.49	[1.71–13.27]	**0.012**
EQ-5D			
Score	19.23	[10.55–27.91]	**≤0.001**
VAS	14.93	[7.34–22.53]	**≤0.001**
CESAM score	33.86	[18.22–49.50]	**≤0.001**

* *calculated form the formula ((M3-M0) / ((M3+M0)/2)) x 100 and expressed in percentage; β, Coefficient of regression beta; CI, confident interval; DSSI, Duke Social Support Index; WEMWBS, Warwick-Edinburgh Well-being scale; EQ-5D, EuroQol-5D; VAS, Visual analogic scale; CESAM, Center of Excellence Self-AdMinistered questionnaire; The bold values indicate the significant values of p < 0.05*.

## Discussion

The findings of this RCT show that the 3-month cycle of weekly virtual MMFA tours had multidimensional benefits in participating older adults. Social isolation decreased and both physical and mental health improved significantly.

The decrease in social isolation reported in our study is consistent with the results of previous studies, which have shown that arts-based activities can reduce social isolation, and that these interventions are most effective when they are practiced in a group setting and actively engage participants (10–13). In addition, a meta-analysis previously demonstrated that interventions that focus on changing a person's perceptions and that stimulate positive emotions are more beneficial than those that focus on building social ties (15). Furthermore, interventions involving cultural activities, such as the visual arts, regardless of artistic genre or type of activity, have demonstrable benefits including the generation of positive emotions, which have been shown to improve wellbeing, self-esteem and quality of life (16, 17). We suggest that it is for all these reasons that we observed significant social and health benefits in our RCT.

Social isolation is a major problem in Canadian society. The proportion of Canadians aged 65 and over who report experiencing social isolation is high: in 2018, it was estimated to be around 20% of the older population, representing 1.5 million people (27, 28). Social isolation in combination with health challenges, which are often prevalent as we age, expose older individuals and the wider community to a variety of adverse outcomes with deleterious effects (5, 6). For instance, lack of contact between members of a family or within society may hamper or break down intergenerational relationships, increasing feelings of social isolation (29, 30). The physical and mental health issues known to arise as a result of social isolation may increase people's needs for health and social services, which puts pressure on those who work in these systems. This may in turn increase service expenditures (5, 6). Indeed, in 2016, the International Federation on Aging reported that “*the main new problem facing seniors in Canada is maintaining their social contacts and activities*” (31). This highlights the need for effective interventions that promote the social inclusion of older adults *before* they experience social isolation. The 3-month cycle of weekly virtual MMFA tours examined in our study seems to be one example of an intervention that effectively created social connection, the opposite of social isolation.

Many museums offer participatory arts-based activities (19, 32–37). The United Kingdom was one of the first countries to consider museums as partners in social and health policy. This gave rise to a consortium known as National Alliance for Museums, Health and Wellbeing, “(2015-2018), which became a driving force in the British Ministry of Health and Social Services ^1^ and is now known as the Culture, Health and Wellbeing Alliance. The interventions offered in British museums are most often interactive and participatory group activities ^1^. In the same period in Canada, the MMFA began developing participatory, arts-based activities in 2015 (19). Like their British counterparts, the MMFA focused on participatory, art-making workshops, for which improvements in the quality of life and wellbeing of community-dwelling older adults, as well as a reduction in their physical frailty, were reported (19, 31, 32). Our RCT reproduces and confirms previous studies that demonstrate the benefits of thoughtful, interactive, participatory arts-based programming on the physical and mental health of older adults interested in art and culture. The observation that arts-based activities can be beneficial to physical and mental health is not a new one, as exemplified by the field of art therapy (32–35). Improvements in wellbeing and quality of life have been reported in patients with cancer, neuropsychiatric diseases, or physical disabilities (32, 35). Unlike previous studies, this investigation of the MMFA and the guided tours that they developed during the COVID-19 pandemic is the first time, to the best of our knowledge, that these benefits have been documented and reported on virtual museum tours. Taken together, these findings suggest that arts-based activities, even when delivered online, retain their health benefits for older adults. A causal explanation of these complex health benefits is likely attributable to the dynamic interaction between wellbeing, health-related quality of life and physical health. Indeed, a sequence of health benefits has been suggested in previous studies (19). To summarize, the positive experiences engendered by arts-based activities delivered online may improve wellbeing, which improves quality of life and finally, physical and mental health when they incorporate into this virtual environment the principles mentioned previously: an emphasis on group activities; goal-oriented, purposive endeavors; and activities with a creative component.

The RCT design and the standardization of the 3-month cycle of weekly virtual MMFA tours were the main strengths of our study. However, some limitations need to be considered. First, the RCT was carried out in the older population living exclusively in Montreal. Second, even if benefits were reported for social isolation, physical and mental health, it is not possible to identify and isolate respective causal mechanisms. For example, mental and physical health benefits may result from the break in social isolation experienced because of participants', engagement in the study itself. Third, how much “control” we had over the control group was impossible to monitor with precision. Over the study period, the control group may have been exposed to activities that may have influenced the RCT outcomes. We tried to limit this effect by asking the control group participants to withhold participation any in arts-related interventions and social programs over the study period. No participants in the control group reported arts-related or social program activities, however, it was beyond our mandate to monitor. Fourth, there were significant differences between the intervention and control groups' baseline characteristics. In both groups there was a high proportion of females. However, this proportion differed significantly, with fewer females in the intervention group. Sex is a biological characteristic that may differentially impact the outcomes assessed in our RCT. Furthermore, there were also fewer Caucasians in the intervention group and this difference in ethnicity also could affect the results. However, it should be noted that all linear regression models were adjusted based on these baseline characteristics in order to limit their impact.

## Conclusion

Our RCT suggests that a 3-month cycle of weekly virtual MMFA tours may decrease social isolation, foster a sense of connectedness and, thereby, improve mental and physical health in community-dwelling older adults. Like other arts-based activities, this particular program, delivered online, appears to have been an effective digital cultural intervention to mitigate social isolation and the progression of physical frailty, positioning museums as key stakeholders for social and health prevention, and for fostering social connectedness, in the aging population.

## Data availability statement

The datasets used and analyzed in the current study will be made available by the corresponding author upon reasonable request. Requests should be sent to the corresponding author: OB, PhD; Research Centre of the Geriatric University Institute of Montreal, Montreal, QC, Canada; olivier.beauchet@umontreal.ca. All requests need a cover letter explaining the objective, justification, and referent Ethics Committee.

## Ethics statement

The study received approval from the CIUSSS Centre-Sud-de-l'Île-de-Montréal (Quebec, Canada) Research Ethics Committee approved the study (# 2022-1338 – CÉR VN 21-22-08). The patients/participants provided their written informed consent to participate in this study.

## Author contributions

OB: principal investigator, study conception and design, obtaining funding, drafting the manuscript, revision of the manuscript, and final approval of the manuscript. JM, KG, CL, and KS: drafting the manuscript, revision of the manuscript, and final approval of the manuscript. All authors contributed to the article and approved the submitted version.

## Funding

This trial was funded by *Fonds de Recherche du Québec Société et culture*; *Actions concerté*es / *Action sur le vieillissement actif de la population au Québec* / *Projet de recherche-action* – Project 281107. The funding source had no role in the design of the nor on execution, data management, analyses, interpretation, or publication of the results.

## Conflict of interest

The authors declare that the research was conducted in the absence of any commercial or financial relationships that could be construed as a potential conflict of interest.

## Publisher's note

All claims expressed in this article are solely those of the authors and do not necessarily represent those of their affiliated organizations, or those of the publisher, the editors and the reviewers. Any product that may be evaluated in this article, or claim that may be made by its manufacturer, is not guaranteed or endorsed by the publisher.

## Footnotes

^1^ https://www.google.ca/search?sxsrf=ACYBGNTU3BtvG3NM3zq7ZcuxogmZxn45qA%3A1580848264124&source=hp&ei=iNQ5XreMBfCh_Qb4pb-YAg&q=2018+English+Alliance+of+Museums+for+Health+and+Wellbeing&oq=2018+English+Alliance+of+Museums+for+Health+and+Wellbeing&gs_l=psy-ab.3.0.0.1306.9211.0.12174.0.0.6.0.0.0.124.745.10j1.0.0.0.0.0.0.0.0.0.1j2..gws-wiz.0.0.0.0.0.0.0i131j0j35i39j0i22i30j0i22i10i30.z4JigKYWf4U&ved=0ahUKEwi38OXZ3rjnAhXwUN8KHfjSDyMQ4dUDCAs&uact=5#spf=1580848276734

^2^ https://www.blueprintincome.com/tools/life-expectancy-calculator-how-long-will-i-live/

## Abbreviations

ADL: Activities of daily living
CRIUGM: Research Center of the Geriatric University Institute of Montreal
CESAM: Center of Excellence Self-AdMinistred questionnaire
DSSI: Duke Social Support Index
EQ-5D: EuroQol-5D
IADL: Instrumental activities of daily living
MMFA: Montreal Museum of Fine Arts
VAS: visual analogic scale
WEMWBS: Warwick-Edinburgh Mental Wellbeing Scale.
